# Preprocedural Imaging Review Before Performing Epidural Steroid Injections: Analysis of Physician Practice Parameters

**DOI:** 10.3390/diagnostics15060729

**Published:** 2025-03-14

**Authors:** Jamal Hasoon, Aila Malik, Christopher L. Robinson, Grant H. Chen, Jatinder Gill

**Affiliations:** 1 Department of Anesthesiology, Critical Care, and Pain Medicine, The University of Texas Health Science Center at Houston, Houston, TX 77030, USA; 2 Department of Physical Medicine and Rehabilitation, The University of Texas Health Science Center at Houston, Houston, TX 77030, USA; 3 Department of Anesthesiology, Perioperative, and Pain Medicine, Harvard Medical School, Brigham and Women’s Hospital, Boston, MA 02115, USA; 4 Department of Anesthesiology, Critical Care, and Pain Medicine, Harvard Medical School, Beth Israel Deaconess Medical Center, Boston, MA 02215, USA

**Keywords:** epidural steroid injection, safety, interventional pain management, chronic pain, radiculopathy

## Abstract

**Introduction:** Epidural steroid injections (ESIs) are a common interventional treatment for managing spinal pain complaints. Despite their widespread use, practice patterns among physicians performing ESIs vary significantly. This study aimed to evaluate preprocedural imaging review by pain physicians who perform ESIs in the cervical, thoracic, and lumbar spine. **Methods:** A survey was distributed to a cohort of physicians who regularly perform ESIs. The survey comprised questions regarding preprocedural imaging review before performing ESIs in the cervical, thoracic, and lumbar spine. The respondents included a diverse group of pain management physicians from various specialties and practice settings. **Results:** The results revealed that the majority of interventional pain management physicians personally interpret their own imaging, followed by a significant percentage of physicians who rely on the radiology reports. There were no physicians who did not perform any imaging review prior to ESIs. Whereas all respondents reported some form of imaging review, only 63.86%, 53.75%, and 64.44% reviewed the actual images prior to cervical, thoracic, and lumbar access, respectively. **Conclusions:** This survey provides initial data regarding imaging reviews among physicians who perform ESIs. Our results demonstrate that physicians treat imaging review as an essential component of the preprocedural process for performing ESIs, as all physicians reported that they perform some form of imaging review before performing ESIs. However, there is only partial adherence to the multidisciplinary working group opinion that segmental imaging should be reviewed for adequacy of space prior to cervical epidural access.

## 1. Introduction

Epidural steroid injections (ESIs) are a common interventional treatment for managing pain related to spinal pathologies, such as disc herniations, spinal stenosis, and degenerative disc disease causing central or foraminal stenosis [1,2]. These procedures aim to deliver corticosteroids into the epidural space to reduce nerve root irritation and provide pain relief. The epidural space is an anatomical area situated between the dura mater and the ligamentum flavum dorsally and between the dura mater and the posterior longitudinal ligament ventrally. Laterally, the space opens into the neural foramen. Variations in spinal anatomy can significantly influence the effectiveness and safety of ESIs. Anatomic anomalies, such as spinal stenosis, transitional anatomy, prior surgery, or scoliosis, can complicate needle placement and increase the risk of complications. Despite their widespread use, ESIs are not without risks, including dural puncture, infection, epidural hematomas, nerve damage, or even neurologic injury and death [3,4].

A preprocedural imaging review is a critical step in minimizing procedural risks and improving patient safety [5]. Imaging allows for a detailed assessment of the spinal anatomy, helps identify contraindications, and provides valuable insights into needle trajectory planning. However, variability exists in clinical practice, with some physicians relying solely on radiology reports rather than personally reviewing imaging studies.

We developed a survey to evaluate the practice parameters of interventional pain physicians who perform ESIs, including questions regarding their preoperative imaging review before performing ESIs in the cervical, thoracic, and lumbar spine. The purpose of this paper is to discuss the importance of preoperative imaging reviews before performing ESIs, highlighting their impact on patient safety, procedural accuracy, and overall efficacy, and share the results of our survey of pain physicians who regularly perform epidural steroid injections.

## 2. Methods

A survey covering various practice parameters of physicians performing epidural steroid injections was developed. This survey was designed based on the perceived clinical importance and interest within the interventional pain community. The survey was developed by a panel of board-certified interventional pain physicians with expertise in performing ESIs. The questions were designed to capture current practice patterns regarding preprocedural imaging reviews before performing ESIs at the cervical, thoracic, and lumbar levels. The initial draft of the survey was reviewed by a small group of academic pain management specialists to ensure clinical relevance and comprehensiveness. Modifications were made based on their feedback to improve clarity. The final version of the survey was reviewed and approved by the Institutional Review Board (IRB) at The University of Texas Health Science Center at Houston.

The survey was distributed over a three-month period (1 March 2024–31 May 2024) via email, as well as through online social media groups, using an online survey platform. Physicians in both academic and private practice settings were invited to participate. Participation was voluntary and anonymous, and completion of the survey implied informed consent from the respondents. The initial page of the survey described the nature of the study, including the intent to collect data for publication, and served as the informed consent statement. Physicians who did not wish to participate could close the survey without proceeding. Similarly, physicians who do not perform ESIs could exit the survey without starting. To prevent duplicate responses, the survey link was restricted to one submission per device.

The inclusion criteria were physicians who regularly perform ESIs in clinical practice. The exclusion criteria included physicians who do not perform ESIs or those who did not continue to the survey.

Due to the wide range of topics covered by the survey, important clinical aspects were grouped and will be published separately. Here, we present our findings on preprocedural imaging reviews prior to performing ESIs. The three questions related to this were as follows:

1. Before performing LUMBAR epidural steroid injections for pain, I review the following.
2. Before performing CERVICAL epidural steroid injections for pain, I review the following.
3. Before performing THORACIC epidural steroid injections for pain, I review the following.

## 3. Results

The survey results for the three questions are presented below. Survey responses were received between 1 March 2024 and 31 May 2024. The survey was sent through email to academic physicians in ACGME-approved fellowship training programs, as well as through social media to physicians who practice interventional pain medicine. Since the survey was distributed across various platforms, including email, web links, and social media, we can only report on those who initiated and completed the survey, making it impossible to determine an accurate response rate. Overall, we believe that these findings offer valuable preliminary data on practice parameters for interventional pain physicians.

**Figure 1:**
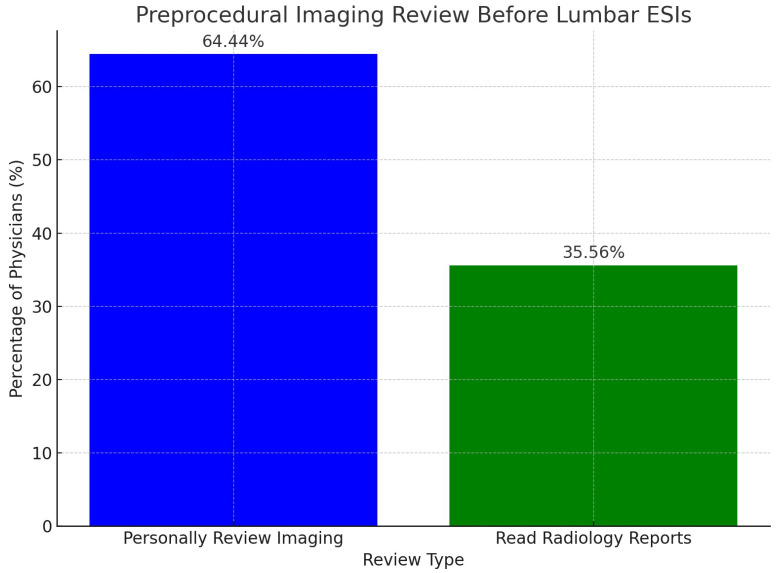
This bar graph illustrates the distribution of practices among physicians regarding preprocedural imaging reviews before performing lumbar epidural steroid injections (ESIs).

When evaluating preprocedural imaging reviews before lumbar ESIs, 64.44% (58/90) of physicians personally interpreted and reviewed all relevant imaging before performing lumbar ESIs. This was followed by 35.56% (32/90) of physicians who indicated that they read the radiology reports before performing lumbar ESIs. There were no physicians who reported that they do not review any relevant imaging or reports before performing lumbar ESIs. These results demonstrate that all responding physicians either personally review relevant patient imaging or at least read the radiology reports before performing lumbar ESIs. Of note, a total of 90 physicians completed this question, with 6 physicians skipping this question. The results are displayed in Figure 1 below.

**Figure 2:**
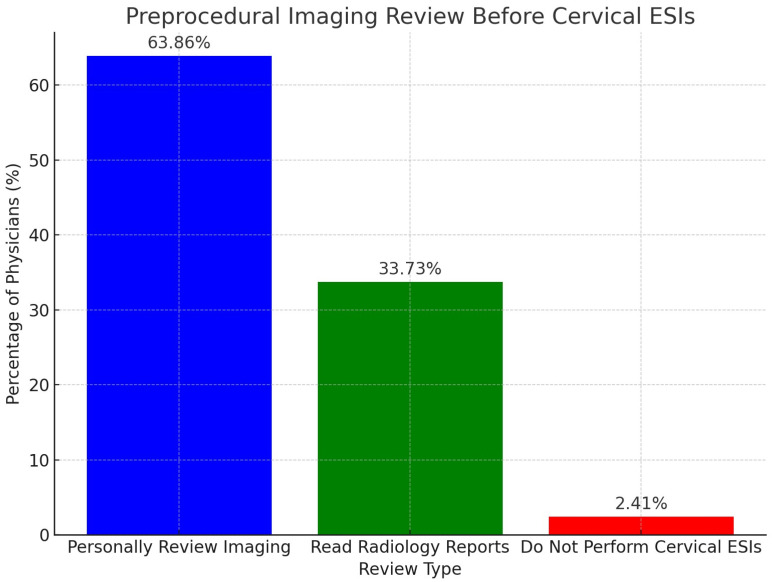
This bar graph displays the practices of physicians regarding preprocedural imaging reviews before performing cervical epidural steroid injections (ESIs).

When evaluating preprocedural imaging review practices before cervical ESIs, 63.86% (53/83) of physicians personally interpret and review all relevant imaging before performing cervical ESIs. This was followed by 33.73% (28/83) of physicians who read the radiology reports before performing cervical ESIs. There were no physicians who reported that they do not review any relevant imaging or reports before performing cervical ESIs. Of note, 2.41% (2/83) of physicians responded that they do not perform cervical ESIs. These results demonstrate that all responding physicians who perform cervical ESIs either personally review relevant patient imaging or at least read the radiology reports before performing cervical ESIs. Of note, a total of 83 physicians completed this question, with 13 physicians skipping this question. The results are displayed in Figure 2 below.

**Figure 3:**
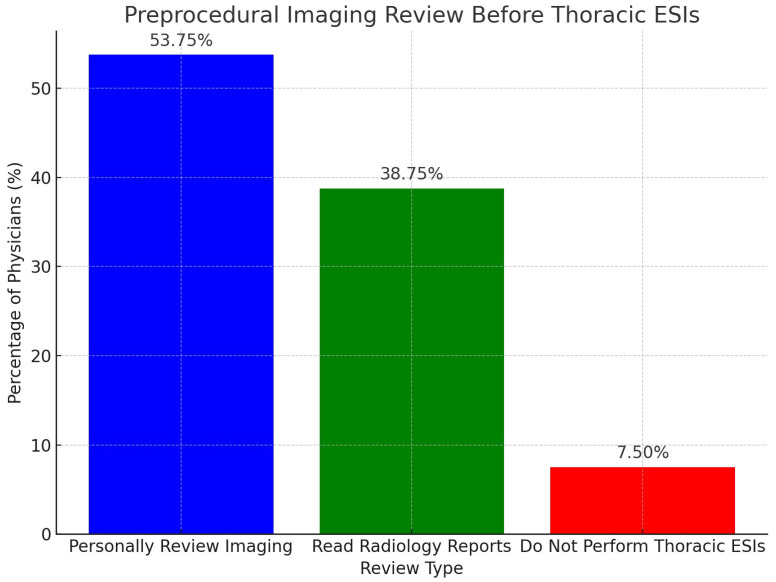
This bar graph displays the practices of physicians regarding preprocedural imaging reviews before performing thoracic epidural steroid injections (ESIs).

When evaluating preprocedural imaging review practices before thoracic ESIs, 53.75% (43/80) of physicians personally interpret and review all relevant imaging before performing thoracic ESIs. This was followed by 38.75% (31/80) of physicians who read the radiology reports before performing thoracic ESIs. There were no physicians who reported that they do not review any relevant imaging or reports before performing thoracic ESIs. Of note, 7.5% (6/80) of physicians responded that they do not perform thoracic ESIs. These results demonstrate that all responding physicians who perform thoracic ESIs either personally review relevant patient imaging, or at least read the radiology reports, before performing this procedure. Of note, a total of 80 physicians completed this question, with 16 physicians skipping this question. The results are displayed in Figure 3 below.

To better contextualize the findings, we provide details regarding the specialty background and practice settings of the 96 survey respondents who initiated the survey. The majority of participants (75%) were Anesthesiologists, followed by Physiatrists (18.75%), Neurologists (4.17%), Radiologists (1.04%), and Emergency Medicine physicians (1.04%). These specialty distributions highlight that the survey primarily reflects the practice patterns of pain management specialists with backgrounds in anesthesiology and physical medicine and rehabilitation.

Of the 95 respondents who provided information about their practice settings, 52.63% of respondents were academically based physicians, while 18.95% were employed in hospital-based settings. Physicians in private practice employment made up 20% of the sample, and 8.42% were owners or partners in a private practice. These findings indicate that the study captures perspectives from a diverse range of practice environments, although with a predominance of academic practitioners.

No additional demographic information was collected in the survey. However, understanding the specialty and practice setting of respondents provides valuable context for interpreting the results, as practice patterns may vary based on training background and institutional setting.

## 4. Discussion

ESIs are widely used for managing chronic spinal pain and radicular symptoms associated with conditions such as disc herniation, spinal stenosis, and degenerative disc disease. While ESIs are generally effective and safe, their success and safety can be enhanced by conducting a review of the preoperative imaging. This is especially critical in patients with a complex spinal anatomy or a history of prior spinal surgery. Preoperative imaging helps clinicians navigate challenges related to complex anatomies and tailor their therapeutic approach with regard to the targeted vertebral level and injection technique. Below, we briefly discuss the importance of a preoperative imaging review and its use in specific patient populations of interest in the context of our study findings.

### 4.1. Benefits of Preoperative Imaging Review

#### 4.1.1. Enhanced Diagnostic Accuracy and Procedural Planning

A preoperative imaging review significantly enhances the diagnostic accuracy by providing a comprehensive assessment of the spinal pathology. When used in conjunction with a thorough clinical examination, detailed imaging may allow clinicians to better identify the source of pain, distinguish between different types of spinal disorders, and assess the severity of the condition. This information is important for determining the appropriateness of ESIs and selecting the most suitable candidates for the procedure.

Preoperative imaging is also crucial for procedural planning by providing detailed anatomical maps. This is especially important in complex cases, where anatomical variations, such as altered spinal alignment, transitional anatomy, or previous surgical alterations, are present. A comprehensive review of the imaging can help clinicians determine the optimal injection site, angle of needle insertion, and volume of injectate. Detailed imaging helps in planning the safest and most effective approach for ESIs, thereby reducing the likelihood of procedural errors and enhancing the likelihood of successful outcomes [5].

#### 4.1.2. Increased Patient Safety

Patient safety is paramount in any medical procedure, and a preoperative imaging review significantly contributes to this aspect. By identifying potential complications, such as vascular anomalies or anatomical changes after surgery, clinicians can modify their approach to avoid these pitfalls. For example, identifying a patient with a disc herniation at the same level as a prior laminectomy, the physician may choose to avoid an interlaminar ESI at that level given the disruption of the anatomy and increased risk of dural puncture. Additionally, imaging can help detect contraindications to ESIs, thereby preventing adverse events. Preoperative imaging can also help physicians to confirm the correct levels before the procedure. This ensures precise localization of pathology and confirms proper needle positioning. Additionally, integrating imaging with the contralateral oblique view enhances injection planning, as studies show this view provides superior visualization over the lateral view for cervical and lumbar epidural access, improving procedural accuracy and patient outcomes [6,7]. Reviewing the imaging can further improve the safety and accuracy of this view by providing an estimation of the laminar angle, as well as an estimation of the ligamentum flavum thickness and expected needle tip location at the time of epidural access [3,8,9].

#### 4.1.3. Optimization of Injection Technique

The success of ESIs largely depends on the precision of the needle placement and the distribution of the steroid solution within the epidural space to the target. Preoperative imaging that matches patient symptoms provides critical information regarding the target for medication placement, which can optimize the injection technique. For instance, in cases of foraminal stenosis or a foraminal disc protrusion, imaging may direct a physician to choose a transforaminal approach, which may provide more targeted relief than an interlaminar injection. Similarly, imaging can help identify the optimal vertebral level for injection to access the target pathology. Procedural contrast spread patterns can then provide evidence of whether the target was accessed. Reviewing imaging can also help make sense of the diverse contrast patterns that may be seen [10,11]. Physicians may alter their level of entry based on the severity of the underlying pathology. The target access has been shown to be important in terms of the outcomes of epidural injections [12,13,14].

### 4.2. Special Patient Populations

#### 4.2.1. Patients with Prior Spinal Surgery

Patients with a history of prior surgery present unique challenges for ESIs due to postoperative changes such as scar tissue, altered spinal alignment, and hardware placement. MRI and CT scans are invaluable in these cases. Preoperative imaging can determine disruptions or alterations in the spinal anatomy, such as in the case of laminectomy or decompression surgeries. It also allows for the visualization of metallic hardware, which is critical in planning the injection path to avoid these structures. In patients with prior lumbar fusion, the fused segments may restrict the accessible epidural space. Preoperative imaging helps in identifying non-fused levels that can be targeted for injection. It also assists in choosing between transforaminal and interlaminar approaches based on the available space, safety, and the specific pathology. A study by Song et al. evaluated anatomical changes in the ligamentum flavum (LF) and epidural space in 34 patients after spinal surgery [15]. Their study revealed that an intact LF was observed in 100%, 75%, and 50% of patients, at two levels above, one level above, and one level below the segment level of surgery. An intact epidural space was observed in only 12% and 30% of patients at the upper and lower vertebral level of surgery, respectively. The thickness of the LF and the cross-sectional area of the epidural space significantly decreased at the levels of spinal surgery [16]. In this patient population, an interlaminar approach at the site of prior lumbar surgery would not be recommended due to an increased risk of complications.

#### 4.2.2. Patients with Transitional Anatomy

A transitional vertebra refers to one that retains features of vertebrae from adjacent spinal segments [13]. Lumbosacral transitional vertebrae (LSTV), which may refer to sacralization of L5 or lumbarization of S1, are relatively common, with a reported prevalence of 10% to 29% [17]. Such transitional anatomy may lead to an error in the correct labeling of vertebral levels, resulting in a procedure or surgery being performed at the wrong level. Furthermore, these congenital variations may carry clinical implications, as the patient can have dermatomal variations or altered function of the lumbosacral nerve roots, which may result in the selection of an incorrect spinal level for the ESI [18]. Back pain associated with lumbosacral transitional vertebrae (LSTV), known as Bertolotti Syndrome, remains a debated clinical entity [17]. Traditionally, LSTV are best characterized by Ferguson radiographs (anteroposterior radiographs with a degree of cranial tilt) [19]. The classification of LSTV may be difficult with MRI due to poor visualization of the thoracolumbar junction, difficulty identifying the lowest rib-bearing vertebral body, and limited distinction between thoracic ribs and enlarged lumbar transverse processes [19]. In patients with variant anatomy, preoperative imaging allows for the accurate enumeration of vertebral segments, and correlation of intraoperative radiographs with preoperative imaging is crucial to avoid interventions at the wrong level. Furthermore, the presence of an LSTV may have an impact on patient outcomes following an ESI. A study reported that patients with LSTV (*n* = 47) experienced reduced improvement in pain scores compared to those without LSTV (*n* = 244) six months following a transforaminal ESI (*p* < 0.01), which may be due to pathologic changes in nearby structures, osteophyte formation, and/or extraforaminal stenosis of the affected nerve root associated with the presence of LSTV [20].

The best protection against procedures at the wrong level is identifying the pathological level and superimposing the mid-sagittal 3D scan to the lateral fluoroscopic view, as well as visually confirming that the needle is placed at the level of the pathology.

#### 4.2.3. Patients with Scoliosis

Scoliosis, characterized by lateral curvature of the spine with a Cobb angle ≥ 10 degrees, has a global prevalence of 0.11–5.2% [21]. Scoliosis results in the distortion of key anatomical landmarks, such as the spinous process that is used to identify the midline and iliac crests, which aid in identifying the level of needle insertion. In cases of uncorrected scoliosis, there is deviation of the midline toward the convexity of the scoliotic curve relative to the spinous processes [22]. As such, the interlaminar space is easier to access with a needle that is oriented toward the convexity of the scoliotic curve. In patients with a history of surgical correction, the surgical site should be avoided, as scar tissue, postoperative adhesions, and bony grafts may hinder needle entry into the targeted space and interfere with the spread of injectate. A needle placement above or below the surgical site may be pursued, although in some cases, extensive distal fusion may restrict access to interspaces. Preoperative imaging with MRI or CT scans can identify the extent of vertebral involvement and provide a detailed assessment of the curvature, its severity, and its impact on the epidural space. This information is crucial for planning the needle trajectory and adjust the technique to navigate the spinal curvature safely.

#### 4.2.4. Patients with Lumbar Spinal Stenosis

Spinal stenosis is a degenerative spine condition characterized by narrowing of the central canal, the lateral recess, or neuroforamen [23]. Lumbar spinal stenosis affects approximately 50% of patients suffering from low back pain, and when symptomatic, it presents as buttock and bilateral leg pain occurring with lumbar extension and relieved with lumbar flexion [24]. The diagnosis is largely based on the presence of signs and symptoms of neurogenic claudication and correlation of the clinical picture with causative imaging findings [25]. MRI of the spine is the standard imaging modality utilized to determine the anatomic location of the stenosis, and its severity may be evaluated with grading systems such as the Lee Grading System, which is based on the assessment of perineural fat obliteration and morphological changes in the nerve root [26]. However, it should be noted that MRI and CT findings have been shown to correlate poorly with symptoms [15].

Per the Journal of the American College of Radiology, an MRI should be obtained in the presence of red flags and in patients who fail to respond to six weeks of conservative management with medications and physical therapy [27]. In patients with severe stenosis, imaging can assess the soft tissue and ligamentous structures and areas of bony overgrowth to help determine the safest needle entry point and trajectory to access the epidural space and minimize the risk of dural puncture or nerve injury. The guidelines from the American College of Physicians and the American Pain Society strongly recommend obtaining MRI or CT imaging for patients with persistent low back pain accompanied by signs or symptoms of radiculopathy or spinal stenosis, especially if they are potential candidates for ESIs [28]. Preprocedural imaging can have an impact on clinical decision making with regard to the targeted spinal level and/or approach to the epidural space.

It is the Consensus opinion from a Multi-Society Working Group for Safeguards that to prevent neurological complications, the cervical epidural space at the intended injection level should be inspected on imaging to assure adequacy of the cervical epidural space to allow for safe needle tip placement [5]. In a closed claims analysis of injuries related to cervical procedures, direct needle trauma was noted to be an important cause of neurological injuries [3]. In stenotic patients, the adequacy of the dorsal epidural space is likely to be compromised and must be confirmed, especially for cervical access. In lumbar access, the decision can be made on a case-by-case basis, as the consequences of advancement into the thecal sac are not as dire; nevertheless, epidural collection may increase pressure upon the neural structures.

In summary, this discussion highlights the critical role of preoperative imaging in enhancing the accuracy, safety, and effectiveness of ESIs for patients with diverse spinal conditions. Preoperative imaging allows for a tailored approach to address anatomical variations, surgical history, and the specific pathology of each patient, ultimately improving the diagnostic precision, procedural planning, and patient outcomes. For complex cases such as those with previous spinal surgeries, transitional anatomies, scoliosis, or spinal stenosis, detailed imaging facilitates an optimized injection technique to maximize the therapeutic benefit of ESIs. These findings underscore the importance of incorporating imaging into clinical workflows to refine patient selection and procedural approaches, aligning with recommendations from major pain societies. Even though cervical epidural access is associated with a risk of direct needle trauma to the spinal cord, and it is recommended that a review of imaging be undertaken prior to epidural access, in this small survey, only 63.8% of the clinicians’ practice included conducting this review. This points to the inconsistency between the opinions of experts and actual practice parameters of clinicians. However, all physicians either personally reviewed imaging or reviewed radiology reports before performing cervical ESIs. No physicians performed cervical ESIs without some form of imaging review.

## 5. Limitations

This study sheds light on the common practice of preprocedural imaging reviews by physicians performing ESIs. However, several limitations must be acknowledged. The survey was distributed via email to pain medicine physicians within large academic hospital systems and shared with private practice physicians through social media via a clickable link, making it difficult to determine an exact response rate. The inability to determine an exact response rate is a recognized limitation of this study due to the distribution method, which included email outreach and social media dissemination. Because the total number of physicians who received the survey link is unknown, a precise response rate cannot be calculated. However, to provide greater transparency and mitigate concerns about survey credibility, we have analyzed and included the number of skipped questions in this survey.

It is also possible that some recipients may not routinely practice interventional pain procedures, potentially affecting the survey’s relevance to all respondents and potentially introducing self-selection bias. Physicians with a greater interest in imaging review may have been more likely to participate, potentially skewing the results toward higher reported rates of image interpretation. Conversely, those who rely primarily on radiology reports or do not emphasize imaging reviews may have been under-represented. This could have resulted in an overestimation of the proportion of physicians who personally review imaging before performing ESIs. Nonetheless, given the survey’s focus on identifying specific practice patterns among physicians who perform ESIs, and considering that the emails and links targeted interventional pain physicians, it is likely that most respondents are actively using this therapy in their clinical practice, minimizing the potential for skewed results.

Despite these limitations, this study provides valuable preliminary insights into physicians’ practice patterns regarding imaging reviews before ESIs. The finding that all respondents reported some form of imaging review reinforces the importance of imaging assessment in clinical decision making.

Overall, while this study highlights key trends in physician practice, the limitations necessitate cautious interpretation of the findings and underscore the need for additional research to validate these results in broader physician populations.

## 6. Conclusions

A preoperative imaging review is an essential component of the process for performing epidural steroid injections. It enhances the diagnostic accuracy, helps target identification for the injectate, improves procedural planning, increases patient safety, optimizes injection techniques, and enhances overall efficacy. It also improves outcomes by enabling preprocedural identification of the target, ensuring precise access during the procedure. Target access has been shown to be important for outcomes. Our survey results demonstrate that the majority of interventional pain management physicians personally interpret their own imaging, followed by a significant percentage of physicians who rely on the radiology reports. There were no physicians who reported that they do not perform any imaging review prior to ESIs. There is only partial adherence to multidisciplinary working group opinions that segmental imaging should be reviewed for adequacy of space prior to cervical epidural access.

## Data Availability

The original contributions presented in this study are included in the article. Further inquiries can be directed to the corresponding author.
